# Post-Hyaluronic Acid Recurrent Eyelid Edema: Pathophysiologic Mechanisms and a Proposed Treatment Protocol

**DOI:** 10.1093/asjof/ojad102

**Published:** 2023-12-03

**Authors:** Justin Karlin, Neil Vranis, Erez Dayan, Kami Parsa

## Abstract

**Background:**

Hyaluronic acid (HA) filler injections for facial augmentation are commonly administered but can lead to post-hyaluronic acid recurrent eyelid edema (PHAREE). The pathophysiology of this condition has not been fully understood.

**Objectives:**

To report the successful treatment of PHAREE using serial hyaluronidase and fractionated radiofrequency microneedling, with additional carbon dioxide laser skin resurfacing in selected patients.

**Methods:**

Five patients with PHAREE were treated with serial hyaluronidase injections and fractionated radiofrequency microneedling, with 2 patients receiving carbon dioxide laser treatment. The patients were followed up for a minimum of 24 months.

**Results:**

All patients reported a resolution of PHAREE signs/symptoms with no adverse effects or recurrence. One patient demonstrated complete resolution after a single treatment; 4 required a series of treatments.

**Conclusions:**

The proposed treatment protocol may provide advantages over hyaluronidase alone for PHAREE. The impermeable malar septum, vulnerable eyelid lymphatics, and potential immunogenicity of HA fragments likely contribute to PHAREE pathophysiology. Further research on pathophysiologic mechanisms is warranted.

**Level of Evidence: 4:**

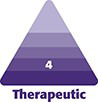

Facial volume augmentation with hyaluronic acid (HA) filler gel injection ranks among the most common aesthetic nonsurgical procedures performed worldwide. One estimate reports that the number of procedures of soft-tissue injection of HA fillers increased from 1.2 million in 2012 to 3.6 million in 2020.^1^ This increase in popularity can be attributed to high patient satisfaction, immediate results, social media marketing campaigns, perceived high safety profile (reversibility^2^ and biocompatibility^3^), and the low barrier to entry (low overhead, quick procedure time, and minimal technical demand). If these factors are coupled with high profit margins and low financial burden (compared with surgical procedures), then the HA filler injection procedure becomes a lucrative adjunct to the practices of facial surgeons, dermatologists, nonsurgical general doctors, dentists, and physician extenders (ie, nurse injectors, nurse practitioners, and physician associates).

As expected, a rise in the frequency of facial HA filler injection procedures portends a corresponding rise in associated complications.^4-12^ These complications can present early or late; be transient, intermittent, or persistent; and include mechanical, inflammatory, or ischemic etiologies. Common nonischemic, noninflammatory complications include contour abnormalities (static or dynamic), skin discoloration, localized excessive tissue expansion, or panfacial “overfill.” Inflammatory nonischemic complications may include mycobacterial infection, foreign body granuloma formation, and facial cellulitis. Complications secondary to vascular compromise^13,14^ have gained significant attention, given the severity and need for emergent medical evaluation. Persistent or intermittent malar and eyelid edema is an underreported complication that is poorly understood^15,16^ but can be quite distressing to both patient and injector. There is a knowledge gap in understanding how post-hyaluronic acid recurrent eyelid edema (PHAREE) develops and a skills gap in how to treat this condition.

PHAREE and related conditions have been associated with a periocular injection of HA fillers. The common sites of periocular HA injection include effacement of the inferior orbital rim hollow,^15^ colloquially referred to as the “tear trough injection,” and augmentation of the midface,^17^ that is, expansion of the suborbicularis oculi fat (SOOF) pocket by injecting filler material into the prezygomatic space. There have been numerous reports of edema of the lower eyelid and midface following HA filler injection.^15,16,18-21^

Key anatomic and histologic features of the lower eyelid and midface can render this region susceptible to short- and long-term complications such as PHAREE. Anatomically, these features include an abrupt transition between the thin skin of the eyelid and the thick skin of the cheek,^22^ which may result in visible irregularities beneath the eyelid skin, with even a slightly superficial injection^23^; variable extent of the arcus marginalis and orbital septum, making inadvertent orbital filler injection possible, worsening rather than camouflaging steatoblepharon; and the orbicularis oculi's sphincter-like muscle orientation, which, in the case of inadvertent intramuscular injection, may result in dynamic irregularities during animation.^23^ Histologically, the contributing factors to PHAREE are the impermeability of the malar septum,^24-26^ which can act as a barrier to retain fluid, especially in the setting of lymphatic damage or the presence of hydrophilic filler material; the eyelid's delicate, superficial valveless lymphatics,^27^ which are vulnerable to damage at the sites of injection (mechanical obstruction secondary to external hydrostatic pressure compression); and the propensity of filler material (especially low-molecular-weight hyaluronic acid and crosslinking agents) to stimulate idiosyncratic inflammation,^28-32^ resulting in a T-lymphocyte-mediated response that may irreversibly damage the lymphatic drainage apparatus, resulting in lymphedema.^33^

Festoons, a related clinical entity,^34-36^ may bear some resemblance to PHAREE but have a different pathophysiologic mechanism. Festoons are characterized by a laxity of the zygomatic-cutaneous ligaments, orbicularis oculi muscle, and overlying skin, a laxity that both leads to and is caused by co-existing tissue edema. Although the exact triggers and pathophysiology of festoons are an active area of research, several treatment modalities have been employed with variable success rates. These include surgical intervention (midface lift and/or lower blepharoplasty),^37^ ablative lasers,^38-40^ chemical peels,^41^ sclerosing agents,^42,43^ radiofrequency (RF) devices,^41^ or a combination thereof.^44^

In this report, we couple the principles and concepts of festoon management with the ability to dissolve the inciting HA filler with hyaluronidase and RF to develop a treatment protocol that can be effective for managing the manifestations of PHAREE. We propose that management should include dissolution of a previously placed filler along with a gradual and controlled retraction of the surrounding soft tissue. A slow and serial dissolution of the filler, while allowing for a synchronous contraction of the soft tissue, prevents skin redundancy and rapid volume loss. Anecdotally, from the experience of the senior author, patients who simply undergo hyaluronidase without simultaneous soft-tissue management often report dissatisfaction because of the presence of deflation and new rhytides. Through this case series, we demonstrate clinical resolution with acceptable aesthetic outcomes of PHAREE in 5 patients. We employ a protocol that combines hyaluronidase (Hylenex; Halozyme Therapeutics, San Diego, CA) injections with fractional RF energy (Morpheus-8; InMode, Irvine, CA) treatments. Carbon dioxide laser (DEKA; Innate Ability, Calenzano, Italy) resurfacing was also performed on 2 patients who demonstrated a low-risk Fitzpatrick skin type.

## METHODS

Five patients with a clinical diagnosis of PHAREE were identified. All included patients reported a history of multiple HA filler injection events to the malar region and orbital rim hollow. The patients initially presented to the senior author's practice during the period between 2018 and 2021. Subsequently, each patient reported bilateral, recurrent, and persistent malar edema. Ultrasound (Clarius, Vancouver, BC, Canada) was performed in all patients to confirm the presence of HA fillers in the prezygomatic, premaxillary, and/or orbital rim hollow region. Demographics, relevant medical history, clinical photographs, and the treatment protocols are presented in Table. All patients were instructed to initiate lifestyle changes, including lower salt diet, facial massage with a jade roller, and sleep hygiene (ie, sleeping on the back and with the head elevated to 15 degrees). After the completion of the individualized treatment plan, all patients were followed up for a minimum of 24 months to assess for recurrence. Written consent was provided, by which the patients agreed to the use and analysis of their data. This study was approved by the Institutional Review Board of the University of California, Los Angeles.

**Table.** Demographics, Relevant Medical History, Clinical Photographs, and the Treatment Protocols Are Presented

Case	Age	HA filler type (if known)	Triggering event (if known)	Treatment regimen overview	Treatment settings
1	52		Autologous fat transfer to the midface	Three rounds of treatment with 30 U hyaluronidase, with concomitant fractionated radiofrequency, 3 to 6 weeks apart	Radiofrequency First pass: depth 2 mm, energy level of 20 Second pass: depth 3 mm, energy level of 25 88 total shots (44 each side)
2	64		Autologous fat transfer	One treatment of 30 U hyaluronidase, with concomitant fractionated radiofrequency and CO_2_ laser skin resurfacing	Radiofrequency First pass: depth 2 mm, energy level of 12 120 total shots (60 each side) CO_2_ laser 10W, dwell time 500 μm, spacing 450 μm
3	54	Juvederm Plus (Allergan, Irvine, CA)	Sculptra injection (Galderma, Dallas, TX)	Two rounds of treatment with 75 U hyaluronidase, with concomitant fractionated radiofrequency	Radiofrequency First pass: depth 1 mm, energy level of 15 Second pass: depth 3 mm, energy level of 25 100 total shots (50 each side)
4	58	Juvederm (Allergan, Irvine, CA), Restylane (Galderma, Dallas, TX), Voluma (Allergan, Irvine, CA)		One treatment of 50 U hyaluronidase with concomitant fractionated radiofrequency and CO_2_ laser skin resurfacing	Radiofrequency First pass: depth 2 mm, energy level of 18 Second pass: depth 3 mm, energy level of 18 100 total shots (50 each side) CO_2_ laser First pass: 6 W, dwell time 500 μm, spacing 450 μm Second pass: 9 W, dwell time 500 μm, spacing 450 μm
5	49			Five rounds of treatment with 30 U hyaluronidase, with concomitant fractionated radiofrequency	Radiofrequency First pass: depth 1 mm, energy level of 25 Second pass: depth 2 mm, energy level of 25 Third pass: depth 3 mm, energy level of 25 90 total shots (45 each side)

## RESULTS

### Case 1

**Figure 1.**
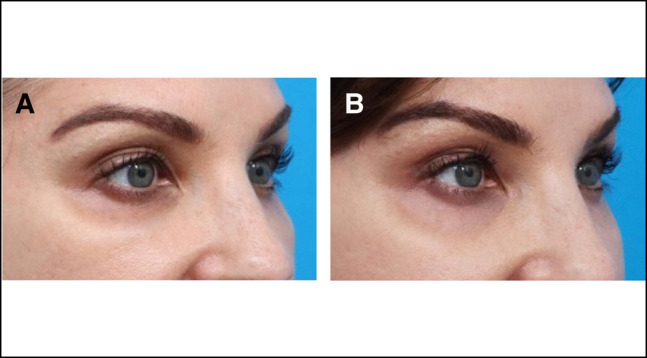
Oblique view of a 52-year-old female patient described in Case 1. (A) Pretreatment photograph and (B) posttreatment photograph. Note the presence of lid–cheek junction edema and blue discoloration of the pretreatment photograph, features that are no longer present in the posttreatment photograph (captured 28 months later).

A 52-year-old woman presented with a history of multiple HA filler injection events to the midface and orbital rim hollow in the 15 years prior to presentation. Five years prior to presentation, she had undergone an autologous fat transfer procedure to the midface by an external plastic surgeon. She developed persistent eyelid and midfacial edema immediately post-procedure. She was referred for the evaluation and treatment of eyelid and malar edema (Figure 1 and Video).

Her treatment regimen included 3 treatments of the following protocol: injection of Hyelenex (hyaluronidase enzyme; Halozyme Therapeutics), 30 units on each side, combined with Morpheus-8 (fractionated RF microneedling; InMode) treatments. A period of 3 to 6 weeks elapsed between each treatment. The concurrent Morpheus-8 treatments included treating the lower eyelids and the midface. The RF treatment settings were as follows: the first pass was set to a depth of 2 mm with an energy level of 20 and the second pass was set to a 3 mm depth and an energy level of 25. The patient received a total of 88 total shots (44 on each side).

### Case 2

**Figure 2.**
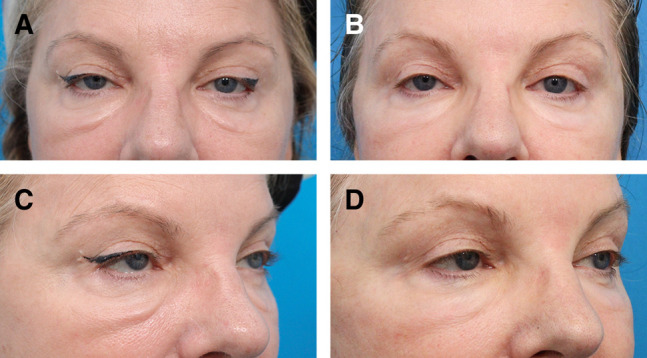
(A, C) Preoperative front facing and oblique photographs of a 64-year-old female (Case 2) presenting with signs of post-hyaluronic acid recurrent eyelid edema and symptoms including intermittent malar edema. (B, D) One year after a single treatment session of enzymatic degradation of a previously placed hyaluronic acid filler, along with fractionated radiofrequency microneedling and carbon dioxide laser skin resurfacing, there was a resolution of symptoms in addition to clinical aesthetic improvement.

A 64-year-old female presented with a 3-year history of intermittent, recurrent, and painless edema of her malar region (Figure 2). She reported a history of filler injection to the lower eyelid orbital rim hollow 5 years prior to presentation. The edema had developed immediately after lower eyelid fat transfer 3 years before presentation.

The patient was treated with 1 round of Hylenex (Halozyme Therapeutics) injection, 30 units on each side, and Morpheus-8 (InMode), with concomitant carbon dioxide laser skin resurfacing. The RF treatment was performed at 2 mm depth with 120 shots (60 per side) in the periocular region. A single pass of fractionated carbon dioxide laser skin resurfacing was performed at a power of 10 W, dwell time of 500 μm, and spacing of 450 μm.

### Case 3

A 54-year-old female presented with 4 years of malar edema. She reported a history of HA filler injection to the midface and orbital rim hollow 5 years prior to presentation. She stated that the edema had first developed 4 years prior to presentation, immediately following a facial injection of Sculptra (Galderma, Dallas, TX).

**Figure 3.**
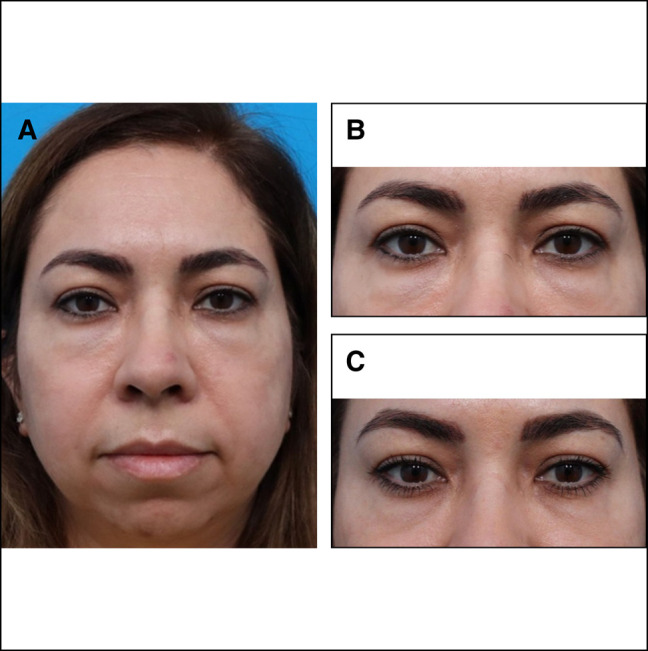
(A) Full-face photograph of a 54-year-old female prior to post-hyaluronic acid recurrent eyelid edema treatment showing chronic swelling over the orbital rim hollows and malar region. Focused periorbital photographs of the same patient, (B) before and (C) 6 months after treatment with hyaluronidase and fractionated radiofrequency microneedling treatment outlined in Case 3.

The patient was treated with 2 rounds of injection of Hylenex (Halozyme Therapeutics), 75 units, coupled with simultaneous fractionated RF microneedling (Morpheus-8; InMode) to the midface with the following settings: 1 mm depth and an energy level of 15 and 3 mm depth and energy level of 25. The patient received a total of 100 shots (50 per side; Figure 3).

### Case 4

**Figure 4.**
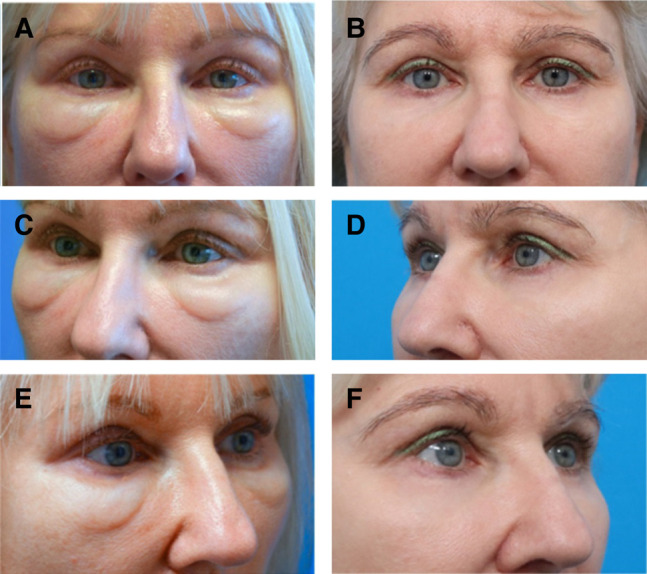
A 58-year-old female with post-hyaluronic acid recurrent eyelid edema was treated with a combination of hyaluronidase, fractionated radiofrequency microneedling, and CO_2_ laser resurfacing (Case 4). (A, C, E) Bilateral oblique and frontal pretreatment views. (B, D, F) The posttreatment photographs were captured 9 months after treatment.

A 58-year-old female with a history of multiple HA filler injections over a 10-year period to the midface and orbital rim hollow presented reporting several years of fluctuating edema of the bilateral lower eyelids. The patient denied experiencing pain and erythema. The edema was noted to be worse in the mornings and exacerbated by the consumption of salty foods or alcohol (Figure 4).

This patient underwent 1 round of treatment with Hylenex (Halozyme Therapeutics), with 50 units injected into the midface and cheek on each side, with concomitant fractionated RF treatment (Morpheus-8; InMode). The RF microneedling settings were as follows: 1 pass at a depth of 2 mm and a second pass at a depth of 3 mm, and at both times, the energy level was set to a power of 18, with a total of 100 shots (50 each side). Additionally, given her favorable skin type, fractionated CO_2_ laser resurfacing (DEKA; Innate Ability) was performed. Two passes were done with the following settings: a power of 6 and 9 W, dwell time of 500 μm, and spacing of 450 μm.

### Case 5

**Figure 5.**
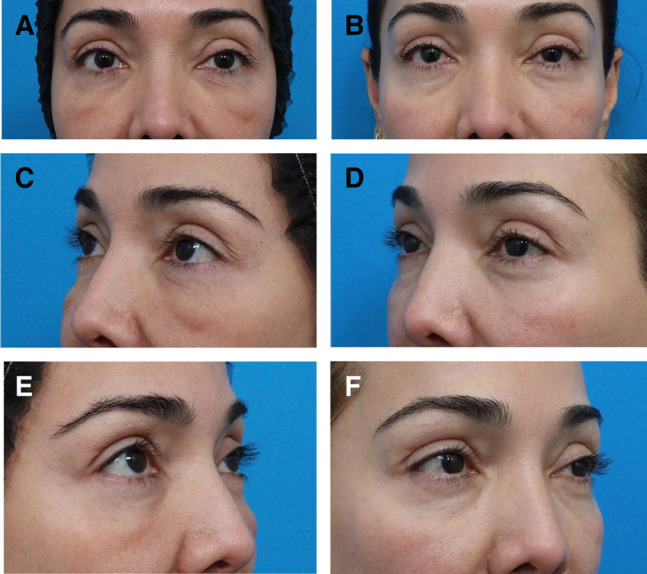
A 49-year-old female with post-hyaluronic acid recurrent eyelid edema was treated with a combination of hyaluronidase injection and fractionated radiofrequency microneedling. (A, C, E) Pretreatment bilateral front facing and oblique views. (B, D, F) The posttreatment photographs were captured 1 year after treatment.

A 49-year-old female presented for the evaluation and treatment of “under-eye bags,” which had worsened considerably in the month prior to first presentation. She noted fluctuation in severity but could not identify any triggers. She reported a history of multiple episodes of HA filler injection to the cheek and orbital rim hollow, at 10 years, 5 years, and 1 year prior to presentation (Figure 5).

She underwent a series of 5 treatments with the following protocol: injection of 30 units of Hylenex (Halozyme Therapeutics) to the orbital rim hollow region on each side, followed immediately by fractionated RF treatment (Morpheus-8; InMode) to the orbital rim and malar region. The RF microneedling settings were as follows: 1 pass at a depth of 1 mm, a second pass at a depth of 2 mm, and a third pass at a depth of 3 mm; the energy level was set to a power of 25 for all passes.

### Summary of Results

All 5 patients were females with an average age of 55.4 years (range 49-64). All included patients experienced a resolution of the signs and symptoms of PHAREE and all of them reported satisfaction over the treatment provided. Resolution was determined by clinical examination from a board-certified oculoplastic surgeon and upon a review of standardized medical photography that demonstrated a smooth lid–cheek junction transition. Additionally, subjective reports from each of the patients emphasizing the resolution of the intermittent eyelid and malar edema confirmed these clinical observations. The efficacy of treatment lasted a minimum of 24 months after the final treatment (follow-up time averaged 26 months with a range of 24-28 months). There was no recurrence of malar edema and no complications of treatment were observed. Two patients experienced resolution after a single treatment and 3 patients required multiple treatments (range 2-5 treatments). Notably, 3 patients reported that the edema had first developed immediately after a facial procedure was performed in the setting of a previously injected HA filler to the same area. In 2 patients, the triggering procedure was autologous fat injection, and in another, it was an injection of Sculptra (Galderma) triggered PHAREE.

## DISCUSSION

In this case series, we report the cases of 5 patients with PHAREE who were treated with serial hyaluronidase injection and fractionated RF, and, out of these, concomitant carbon dioxide laser skin resurfacing was performed in 2 patients. All these patients experienced a resolution of the recurrent malar and eyelid edema, and all expressed satisfaction with the treatment. We review the literature examining HA-filler-related eyelid and malar edema, discuss the pathophysiology of this entity, and describe the principles underlying our proposed treatment protocol.

### Review of Prior Reports of HA-Filler-Related Eyelid and Malar Edema

HA fillers have been in use as an injectable agent for facial volume augmentation for 25 years. In this time, several groups have noted the clinical patterns of eyelid and midfacial edema. Goldberg and Fiaschetti^15^ described the results of cheek, eyebrow, and zygomatic/septal-confluence/orbital rim hollow HA filler injection in 120 patients. In this report, 15% of patients (*n* = 18) reported “fluid buildup” in the malar region. The authors describe this edema as having particular features—“cold” inflammation occurring in the malar region and often persisting for weeks to months. This is identical to what has been observed in patients with PHAREE. The authors in that study speculate that this edema might be related to lymphedema, and that patients with pre-existing malar triangle edema were at a higher risk for developing this complication.

Griepentrog et al^16^ reviewed the charts of 51 patients who underwent periocular lower eyelid HA filler injection and noted 12 patients (∼24%) who demonstrated prolonged periorbital edema. The authors note that the observed periorbital edema lacked inflammatory features. Of note, the majority of patients (10/12, 83%) in that series with periocular edema had received Juvederm (Allergan, Irvine, CA) injections, and 3 patients (3/12, 25%) reported a history of seasonal allergies. The authors speculate that the increased hydrophilicity of Juvederm may have been responsible for the tendency of patients who received this filler to develop edema.

Prior case reports have described delayed onset eyelid edema after HA filler injection. Iverson and Patel^21^ described a patient who had developed eyelid edema 1 year after HA filler injection. Dubinsky-Pertzov et al^20^ described 17 patients who had developed upper eyelid and brow edema 6 to 24 months (mean 13.8 months) after upper eyelid or brow filler injection.

### The Anatomic and Pathophysiologic Basis of Posthyaluronicacid Recurrent Eyelid Edema (PHAREE)

#### Valveless Lower Eyelid Lymphatic System

The lymphatic anatomy of the eyelid and midface is potentially relevant to PHAREE in 2 key ways. First, histologically, the superficial cutaneous lymphatics are a sparse and delicate network that for the most part lack intraluminal valves.^27^ Second, periocular lymphatic drainage in the region of the malar septum forms a watershed region (Dayan and Parsa, unpublished data, 2023).

The lymphatic system is a generally unidirectional network of vessels that collect and transmit interstitial fluid from the peripheral tissues to the central venous system. Interstitial fluid of the lower eyelid and midface, deposited through a leak from the lymphatic capillary bed, is collected through an “oak leaf” network of lymphatic absorbing capillaries that, at their tips, harbor a valve that regulates fluid entry. The fluid that enters the lumen is transported through a paranasal superficial network that extends from the medial canthus to the submandibular nodes and a lateral superficial network from the lateral canthus to the preauricular nodes. There is also a deep network laterally that drains into the preauricular parotid nodes.

Focusing on the histology of the superficial vessels, in order of increasing luminal diameter, we find that the superficial network consists of 3 interconnected plexuses—the (1) dermal capillary and (2) dermal precollector vessels, and (3) a subcutaneous collector vessel network. Plexus (1) and (2) are valveless and drain into (3) which has valves. Given the lack of intraluminal valves, cutaneous superficial lymphatics are unique in that they may be subject to multidirectional flow depending on pressure gradients. A compromise of the lymphatic drainage distal to the superficial network (for instance with HA filler injection) could increase intraluminal pressure, resulting in the slowing or reversal of interstitial fluid clearance from the eyelid skin. Ultimately, downstream subacute or chronic effects of this dysfunction lead to increased dermal and subcutaneous thickness.

The deep network begins when channels penetrate the orbicularis to connect Plexus 3 (see above) to a deep network of valved lymphatic vessels.^45^ This deep network courses inferolaterally, penetrating the orbicularis-retaining ligament along the orbital rim and entering the deep SOOF. These channels then traverse the superior portion of the prezygomatic space and, at the level of the zygomatico-cutaneous ligament (“McGregor's Patch”), penetrate deeper into the preperiosteal fat layer near the origin of the zygomaticus. These channels continue laterally at this level, ultimately draining into the preauricular nodes within the parotid. Notably, there is a connection between the tarsal conjunctival lymphatics with the superficial networks and the lateral deep network through channels that penetrate the tarsus.

**Figure 6.**
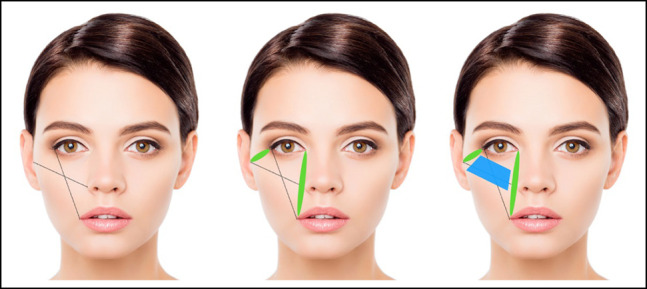
Watershed area of the midface and lower eyelid. The ovals depict the location of the lymphatic drainage pathways serving the lower eyelid and malar region, spanning from the lateral canthus toward the preauricular nodes, and from the medial canthus toward the submental nodes. The trapezoid highlights the watershed region of the midface and lower eyelid.

Conventional descriptions of the lower eyelid and midface lymphatic networks describe a medial network traveling in the paranasal region toward the submandibular nodes and a lateral network traveling along the body of the zygoma toward the preauricular nodes (Figure 6). In this model, as these 2 lymphatic networks diverge toward their nodes (submandibular and preauricular, respectively), a watershed region of the midface forms between the 2 networks. This watershed region happens to correspond to the central and lateral portions of the malar septum, the region of fluid accumulation appreciated in patients with PHAREE.

#### Relatively Fluid-Impermeable Midface Osteocutaneous Septa

The development of PHAREE may be related to the intrinsic anatomy of the lower eyelid and midface. Pessa and Garza described a fibrous septal structure, the “malar septum,” as a “relatively impermeable membrane … that traps tissue fluid and hemoglobin pigment and acts as a functional and structural barrier.” This fibrous thickening spans the lower eyelid and midface, separating the oral cavity and lower face from the orbit and the upper face.^26^ This structure originates at the inferomedial orbit in the region of the anterior lacrimal crest, and fans out laterally, with attachments along the anterior inferior orbital rim (ie, the orbicularis-retaining ligament), and inferolaterally (ie, the zygomatico-cutaneous ligament), with strong attachments at the inferolateral tip of the body of the zygoma, in the region of “McGregor's patch.” The malar septum separates the SOOF into superficial and deep layers. The development of lower eyelid festoons and also the appearance of triangular malar mounds are to some extent related to the unique anatomy of this region,^15^ because osteocutaneous malar septum attachments are not uniform across this anatomic area. Skin laxity or edema will thus result in the development of troughs, in which osteocutaneous attachments are dense, and crests, in which there are no attachments.

**Figure 7.**
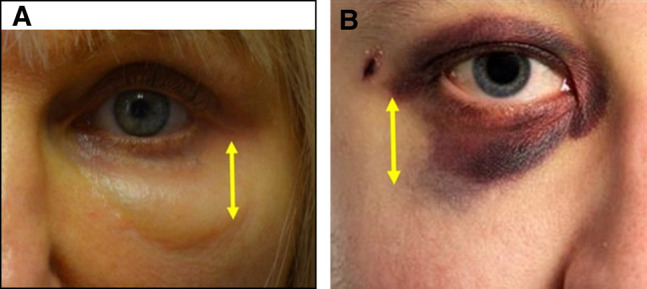
The barrier function of the malar septum. The left image demonstrates periocular ecchymosis and the right image is a closeup of the post-hyaluronic acid recurrent eyelid edema patient described in Case 4. The yellow arrows demonstrate the inferolateral portion of the malar septum. Note the stark transition between (A) ecchymosis and normal tissue and the (B) stark transition between edema and normal tissue (right).

Teleologically, 1 may hypothesize that the malar septum (a fibrous, relatively impermeable structure in the midface) acts as a barrier that isolates the orbit from the oral cavity. The evolutionary benefits of the malar septum may include preventing the translocation of oral pathogens superiorly toward the orbit, which is similar to the orbital septum, which prevents the translocation of the bacterial cellulitis of the face (preseptal) from invading vital structures within the orbit (orbital cellulitis). The barrier function of the malar septum is starkly illustrated in patients who present with periocular ecchymosis, after trauma (Figure 7), in which an abrupt transition corresponding to the thickest attachments of the malar septum exists between the ecchymotic skin of the eyelid and the normal skin of the cheek. Likewise, in patients with PHAREE, the eyelid edema is often noted to accumulate above with an abrupt transition along the lid–cheek junction (zygomatico-cutaneous ligament), at the site of the attachments of the malar septum.

#### Immune Regulation of Lymphedema

An accumulating body of evidence suggests that lymph stasis leads to inflammation, which, in turn, perpetuates permanent damage to the lymphatic system, causing chronic lymphedema. The 3 hallmarks of chronic lymphedema are morphologic adipose deposition, fibrosis, and lymphatic destruction. This process may be regulated by CD4^+^ T cells. In a mouse model of tail lymphedema, CD4^+^ T-cell knockout mice were protected from chronic lymphedema.^46^ In a subsequent paper, the same group identified the proinflammatory cytokine interleukin-6 as a key regulator of adipose deposition in the setting of lymphedema.^47^ Garcia Nores et al showed that lymphatic injury leads to CD4^+^ T-cell activation in regional lymph nodes, and that these T cells migrate to the site of the initial injury.^48^ One might then assume that lymphatic damage creates a pathologic cycle in which migrating CD4^+^ T cells worsen the original lymphatic insult.

Although the exact role of immune-mediated regulation of lymphatic damage in the development of lower eyelid and midfacial edema in the setting of HA injection is still not known, certainly a scenario is possible where HA filler breakdown products might trigger an idiosyncratic T-cell response^49^ that might, in turn, cause lymphatic damage (fibrosis, adipose deposition). This would lead to stasis and, in some cases, permanent lymphatic damage. Similarly, it is possible to imagine a situation where, irrespective of immunologic HA-induced inflammation, a repeated injection of HA fillers to the lower eyelid or midface will cause tissue compression, mechanical lymphatic obstruction, lymph stasis, and localized lymphedema in the delicate superficial lymphatics. These 2 scenarios are not mutually exclusive. Interestingly, in the 2 patients in whom the injection of a non-HA substance triggered PHAREE (Case 1—autologous fat injection, and Case 3—Sculptra), a dissolution of HA fillers and fractionated RF treatment led to PHAREE resolution, suggesting that the presence of HA filler material in certain individuals is necessary but not sufficient to trigger PHAREE.

### PHAREE Treatment Rationale

Injected HA fillers can act as a tissue expander. Goldberg et al^50^ and Zamani et al^51^ have reported using the tissue expanding effect of injected HA fillers to treat lower eyelid retraction, using the fillers to stretch and support soft tissues in 3 dimensions, counteracting eyelid descent. When HA fillers are injected into healthy noncicatrized periorbital tissue, it is reasonable to anticipate that these fillers will have a similar (if not more robust) tissue expansion effect. Modern HA filler products are being manufactured with high levels of crosslinking, in an effort to impart elasticity and enhance product longevity.^52,53^ Products with increased elasticity, by definition, have greater tissue expansion effects. The tissue expander effect of HA fillers, in theory, can stretch native tissues beyond their intrinsic capacitance, causing a fragmentation of elastin and collagen within the dermis and loss of an innate ability to return to baseline.^54^ Following dissolution of the filler, either naturally or deliberately, the overexpanded skin may not return to the elasticity of its prefilled state, leading to a deflated appearance, clinically diagnosed as volume deflation and rhytids. Counteracting deflation is a principle underlying the treatment of PHAREE.

Lymphatic dysfunction plays an integral role in the development of PHAREE. In 1 study, the authors showed that, compared with filler-naive patients (control patients), patients with a history of filler injection to the cheek or orbital rim hollow (or both) had a dysfunctional lymphatic system characterized by a starburst pattern at the lid–cheek junction by near infrared indocyanine green (ICG) lymphoscintigraphy.^55^ The authors observed a localized retention of the dye that lasted for more than 48 h, compared with less than 24 h for the control patients. Interestingly, 2 patients included in the present study (Cases 1 and 3) reported to appear normal after having HA fillers injected to the inferior periorbital area; it was not until after they subsequently underwent a second procedure without dissolving the previously placed filler (Case 1 underwent autologous fat grafting and Case 3 Sculptra injection) that they began experiencing PHAREE. It is possible that in susceptible patients after an initial trigger (ie, HA fillers injected in the cheek, orbital rim hollow, or both), a subsequent inflammatory or injuring insult (fat grafting, lower blepharoplasty, Sculptra, etc) may further disturb the lymphatics beyond a threshold, resulting in PHAREE.

RF microneedling devices can induce remodeling and contraction of dermal and subdermal soft tissues.^56^ A major benefit of RF microneedling is that, unlike ablative lasers that can cause permanent pigmentation changes^57^ in darker-skinned individuals,^58^ these devices can be safely used on patients with higher Fitzpatrick skin types.^59^ A systematic review, which included 2 randomized control trials by Kleidona et al,^59^ affirmed that the use of fractionated RF led to improvements in skin wrinkles, laxity, and patient satisfaction. Note that there have been reports of hyperpigmentation after RF microneedling, but these cases are rare and transient.^60,61^ RF energy delivered to the intra- and subdermal layers induces localized tissue heating, in turn, inducing collagen contraction and collagen synthesis.^62^ RF devices with insulated needles theoretically are able to achieve depth-controlled effects without causing collateral thermal injury to the epidermis or other layers superficial to the desired treatment depth. Fractionated RF devices have been used as a noninvasive modality to improve periorbital rhytids and for the treatment of malar mounds and festoons.^63^ Interestingly, Hsu et al have demonstrated that fractionated RF microneedling can even cause a destruction and dissolution of intradermal and subdermal hyaluronic acid filler material.^64^

Recent data from our group^55^ have demonstrated that patients with HA-filler-related edema demonstrate a delayed clearance of ICG injected into the midface. This is suggestive of slowed lymphatic drainage. Interestingly, ICG clearance improved following the use of hyaluronidase and RF microneedling treatments.^55^ These data support the assertion that hyaluronidase injection, along with fractionated RF, resolves PHAREE by improving lymphatic drainage.

In treating PHAREE, it is first important to determine whether a patient is interested in availing surgical treatment. If so, treatment should involve a combination of HA filler dissolution with hyaluronidase, often with multiple sessions. This should be followed by lower blepharoplasty, possible canthoplasty, and possible midface lift, along with possible simultaneous adjunctive treatments such as fractionated RF microneedling or carbon dioxide laser skin resurfacing or both.

**Figure 8.**
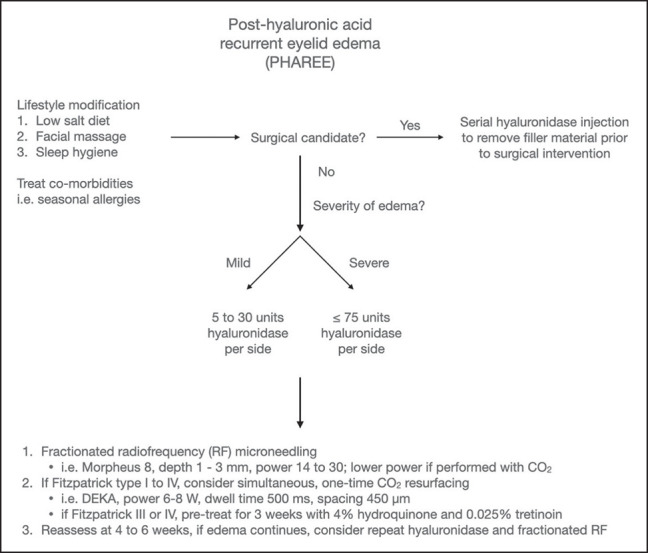
Flowchart outlining the post-hyaluronic acid recurrent eyelid edema treatment protocol. For surgical candidates, the process starts with multiple sessions of hyaluronic acid filler dissolution through hyaluronidase, and then proceeds to surgery. For nonsurgical candidates, the protocol combines low-dose hyaluronidase with fractionated radiofrequency microneedling for gradual filler dissolution and tissue contraction. Optional adjustments include even lower hyaluronidase dosage for mild edema or rhytid prevention and a one-time ablative fractional CO_2_ laser for patients with Fitzpatrick skin Types I to IV. Reassessment every 3 to 6 weeks is essential for nonsurgical candidates.

In PHAREE patients who are not surgical candidates, we believe that it is important to avoid the deflation and tissue laxity that can occur when dissolving the filler using hyaluronidase alone, because this can result in an aged, hollowed appearance. As such, we recommend combining gradual filler dissolution using low doses of hyaluronidase, with synchronous fractionated RF microneedling, to allow for incremental filler dissolution with simultaneous soft-tissue contraction. In patients in whom edema is mild, or in patients who communicate their desire to avoid the formation of rhytids, an even lower dose of hyaluronidase may be injected. Note that fractionated RF microneedling is used not only for its tissue contraction effect, but also for its ability to potentiate the dissolution of HA filler material.^64^ In patients with favorable skin types, a 1-time ablative fractional CO_2_ laser can be incorporated to further optimize tissue contraction, as illustrated in Case 4. Our protocol requires reassessment every 3 to 6 weeks after the initiation of treatment, to determine whether the patient requires further hyaluronidase dissolution with fractionate RF microneedling. Figure 8 is a flow chart describing our treatment protocol in detail.

### Study Limitations

Although all patients in this series reported a resolution of their symptoms, the series’ small sample size limits the generalizability of the findings. Moreover, the lack of a control group (for instance, patients who underwent hyaluronidase injection alone) prevents a comprehensive assessment of the relative contribution of fractionated RF microneedling or laser resurfacing to the treatment of PHAREE. To impart more objectivity and generalizability to the findings, future studies could include standardized outcome measures such as consistent photography with an objective facial edema grading system.

## CONCLUSION

Although the etiology and pathophysiology of recurrent and persistent malar and eyelid edema following HA filler injection are multifactorial, lymphedema plays a major role. In this case series, we have identified and described a therapeutic modality for PHAREE that involves enzymatic degradation of the HA filler, coupled with fractionated RF microneedling (Morpheus-8; InMode), and, in select patients, ablative carbon dioxide laser (DEKA; Innate Ability). We have observed a durable resolution of PHAREE signs and symptoms with a restoration of aesthetic periorbital anatomy, ostensibly due to RF- and laser-mediated tissue contraction and RF-mediated restoration of lymphatic function. Ultimately, managing these rare yet challenging sequelae of HA fillers in the periorbital region with precise multimodal treatments that address the underlying inciting factor, in addition to the overlying soft tissue, results in high levels of patient and surgeon satisfaction.

## Supplementary Material

ojad102_Supplementary_Data
